# Advancing Racial Health Equity Through Family-Focused Interventions for Chronic Disease Management

**DOI:** 10.5888/pcd20.220297

**Published:** 2023-04-13

**Authors:** Katrina R. Ellis, Tiffany L. Young, Aisha T. Langford

**Affiliations:** 1 University of Michigan, School of Social Work, Ann Arbor, Michigan; 2 Lenell and Lillie Consulting, LLC, New Bern, North Carolina; 3 NYU Langone Health, Department of Population Health, New York, New York

The family context has long been regarded as a key setting for health and a target for efforts to strengthen social support for health goals. While improving racial health equity in chronic disease among children and adolescents may more instinctively point toward parent–child experiences, it is not surprising when efforts to improve equity in adults also recognize the role of the family. Broadly speaking, theories and frameworks often bring attention to the family context within considerations of the social environment and social network influences on disease outcomes. In clinic settings, health professionals may speak with adults about their family health history, availability of caregiving, and disease prevention and management within the home environment. Despite these efforts, calls for advancing chronic disease research with families abound. For example, the 2001 Institute of Medicine (IOM) report *Health and Behavior: The Interplay of Biological, Behavioral, and Societal Influences* described family intervention research for chronic disease management among adults as “in its infancy” (1). Deeper attention to the family relationship context, it was argued, was needed to improve chronic disease outcomes for adults. A decade later, the IOM’s 2011 report *Living Well with Chronic Illness: A Call for Public Health Action*, detailed psychosocial, economic, and health-related consequences of chronic illness for families and advocated for greater public health action (2). Moving forward, it is vital that we center racial health equity in our work with adults and their families, including efforts *inside*, *outside*, and *alongside* families.

## Centering Equity: *Inside* Families

The collection and discussion of health history with individuals is where many health professionals regularly engage *inside* families. Indeed, a comprehensive family health history is a valuable tool for assessing risk and determining actions that may enhance health and well-being (eg, start, frequency, and types of cancer screenings; lifestyle changes). Gaps in family history information, particularly by race, hamper these efforts. Innovative tools and creative programs have led to success in improving the completeness of family history collection (eg, collecting information at family reunions) (3). As family history data become more complete, we should ask the question, Is there more we can do with this information? Indeed, there is. Despite its reference to the past, an individual’s family health history can also provide an entree into the extent of family multimorbidity (ie, family members’ co-occurring health issues) and opportunities for family disease management support (4). A family’s experiences with disease management over time have likely led them to develop significant skills and strengths that can be leveraged in intervention efforts. The identification of family-level factors, resources (eg, cooperation, role flexibility), and constraints (eg, conflict, rigid roles) likely associated with disease management are integral to these efforts.

## Centering Equity: *Outside* Families

Interrogating broader sociocultural and contextual factors *outside* of families that shape members’ lives and livelihoods is crucial for equitable intervention design. These factors, and the relationships between them, contribute to family health historically and contemporarily. Over time, families can benefit in some ways and be disadvantaged in other ways by exposure to these inequitable conditions. For example, structural racism influences the community and the built and social environment of families, their ability to access and receive quality preventive and curative care, and their educational and economic opportunities. The distant and recent past is replete with examples of how racism affects the overall health of families. Families often serve as a buffer to racism and discrimination among its members, including providing resources to fortify instabilities resulting from broken, inequitable systems. Families from racially marginalized groups may also take on a greater responsibility to support the health of their members for several reasons, including past experiences with health systems that make accessing services more difficult (eg, poor and/or discriminatory interactions), inadequate treatment when services are provided, and greater disease burden. These caregiving and supportive efforts by families, even when successful, may come at a cost to families’ individual and collective well-being (eg, caregiver burden, network stress).

Care systems in the US are organized such that families are expected to take on varying levels of *responsibility* for the chronic disease welfare of their members. Often lacking with this orientation is attention to variations in the *response-ability* of families (5). What we observe as the ability of families to help their members with the myriad aspects of chronic disease prevention and management is reflective of lifelong and multigenerational embeddedness in inequitable social contexts (6). Thus, our work has to be responsive to the accumulation of advantages and disadvantages across the family life course (7,8) through differential exposures to risk and protective factors in various domains of life. This approach also requires considering the varying levels of health and functioning and the interconnectedness of health and well-being among members of a family unit.

## Centering Equity: *Alongside* Families

Another key component of advancing racial health equity in chronic disease is being committed to working *alongside* families. Individuals are often willing to support their family members in managing chronic illnesses, and there are many organizations and groups at the forefront of these issues. Using community-engaged and participatory approaches to this work is critical. In advocating for an *alongside* approach, Anderson (9) expounds on the importance of balance in working with African American families, arguing for the continued promotion of family “resources and cultural strengths” while simultaneously actively dismantling inequitable and unjust social constraints. In the context of chronic disease prevention and management, this requires tackling the upstream and downstream, proximal and distal factors, long identified as important for chronic disease outcomes.

Reflecting on our disciplinary and personal backgrounds can be useful for building effective partnerships with families we aim to serve while working toward this balance. Hardeman and Karbeah (10) provide a valuable framework for engaging in disciplinary self-critiques that can help us examine how racism has hampered our efforts to achieve health equity. They argue for an examination of our research questions, methodologic approaches, interpretations of our findings, reliance on White-dominant narratives, and what evidence is considered real. These steps toward epistemic justice could also be enhanced by reflecting on additional questions that can help us to identify how personal beliefs, experiences, and biases about family influence our work. For example, how do we define family, personally and professionally, and how might this conceptualization help or hamper our efforts? What do we believe can or should be the role or involvement of families in helping adults manage their health issues? In what ways do we value or promote the needs of the individual over those of the collective (or vice versa)? To what do we attribute the challenges that families such as our own have with managing their health, and how is this similar to or different from the attributions we make about other families? How might our past and ongoing work contribute to narratives about health among the families we serve? Thinking carefully and deeply about these issues can best position us to create meaningful partnerships that can lead to sustainable and practical solutions.

## Conclusion

Inequities in chronic disease outcomes by race in the US are distressing, persistent, and unjust. These inequities have exerted an incalculable toll on generations of families and communities. The policies and practices that will increase racial equity in chronic disease will likely need to be multifaceted and intentional about incorporating a familial approach. Working *inside* families intentionally focuses on family-level factors and processes that influence health outcomes, including concurrent health problems, competing demands of family systems, roles, and relational aspects. Working *outside* families includes bolstering institutional and systemic efforts to redress the social inequities that contribute to disproportionate chronic disease morbidity and mortality rates. Lastly, working *alongside* families includes a commitment to engaging with and partnering with families to design, implement, and evaluate policies and practices designed to improve their chronic disease–related health outcomes. Making progress in complementary *inside–outside–alongside* approaches can lead to positive, synergistic effects that can help families thrive.

## References

[1] Institute of Medicine. Health and behavior: the interplay of biological, behavioral, and societal influences. Washington (DC): The National Academies Press; 2001. p. 221. 20669491

[2] Institute of Medicine. Living well with chronic illness: a call for public health action. Washington (DC): The National Academies Press; 2012.

[3] Hood SM . Enhancing cultural considerations in networks and health: a commentary on racial differences in family health history knowledge and interpersonal mechanisms. Transl Behav Med 2018;8(4):550–3. 10.1093/tbm/iby062 30016526 PMC12104140

[4] Ellis KR , Hecht HK , Young TL , Oh S , Thomas S , Hoggard LS , Chronic disease among African American families: a systematic scoping review. Prev Chronic Dis 2020;17:E190431. 10.5888/pcd17.190431 33416471 PMC7784550

[5] Minkler M . Personal responsibility for health? A review of the arguments and the evidence at century’s end. Health Educ Behav 1999;26(1):121–41. 10.1177/109019819902600110 9952056

[6] Krieger N . Theoretical frameworks and cancer inequities. In: Vaccarella S, Lortet-Tieulent J, Saracci R, Conway DI, Straif K, Wild CP, editors. Reducing social inequalities in cancer: evidence and priorities for research. IARC Scientific Publication No. 168. Lyon (FR): International Agency for Research on Cancer; 2019. p. 111–20.

[7] Bengtson VL , Allen KR . The life course perspective applied to families over time. In: Boss PG, Doherty WJ, LaRossa R, Schumm WR, Steinmetz SK, editors. Sourcebook of family theories and methods: a contextual approach. New York (NY): Springer; 2009. p. 469–504.

[8] Burton LM , Whitfield KE . “Weathering” towards poorer health in later life: co-morbidity in urban low-income families. Public Policy Aging Rep 2003;13(3):13–8. 10.1093/ppar/13.3.13

[9] Anderson LA . Rethinking resilience theory in African American families: fostering positive adaptations and transformative social justice. J Fam Theory Rev 2019;11:385–97. 10.1080/10522158.2019.1681619

[10] Hardeman RR , Karbeah J . Examining racism in health services research: a disciplinary self-critique. Health Serv Res 2020;55(S 2):777–80. 10.1111/1475-6773.13558 32976632 PMC7518806

